# Anticipatory banking of samples enables diagnosis of adenylosuccinase deficiency following molecular autopsy in an infant with vacuolating leukoencephalopathy

**DOI:** 10.1002/ajmg.a.62999

**Published:** 2022-10-22

**Authors:** Spatikha Sitaram, Hetalika C. Banka, Grace Vassallo, Julija Pavaine, Adele Fairclough, Ronnie Wright, Lynette Fairbanks, Jörgen Bierau, Lydia Bowden, Bernd Schwahn, Alistair Horman, Siddharth Banka

**Affiliations:** ^1^ Manchester Centre for Genomic Medicine, St Mary's Hospital Manchester University NHS Foundation Trust, Health Innovation Manchester Manchester UK; ^2^ Department of Paediatric Neurology, Royal Manchester Children's Hospital Manchester University NHS Foundation Trust, Health Innovation Manchester Manchester UK; ^3^ Academic Unit of Paediatric Radiology, Royal Manchester Children's Hospital Manchester University Hospitals NHS Foundation Trust, Health Innovation Manchester Manchester UK; ^4^ Purine Research Laboratory St Thomas' Hospital London UK; ^5^ Department of Clinical Genetics Maastricht University Medical Centre Maastricht The Netherlands; ^6^ Department of Neonatology Royal Oldham Hospital Oldham UK; ^7^ Division of Evolution & Genomic Sciences, School of Biological Sciences, Faculty of Biology, Medicine and Health University of Manchester Manchester UK

**Keywords:** adenylosuccinate lyase, molecular autopsy, reverse phenotyping

## Abstract

Adenylosuccinase deficiency is a rare inborn error of metabolism. We present a newborn who died at 52 days of age with clinical features suggestive of severe epileptic encephalopathy and leukodystrophy of unknown cause. Post‐mortem examination showed an unusual vacuolar appearance of the brain. A molecular autopsy performed via singleton clinical exome analysis revealed a known pathogenic and a variant of uncertain significance in *ADSL* that encodes adenylosuccinase. Tests on previously stored plasma samples showed elevated succinyladenosine and succinylaminoimidazole carboxamide riboside levels. Adenylosuccinase activity in stored fibroblasts was only ~5% of control confirming the diagnosis of adenylosuccinase deficiency in the child. The parents opted for a chorionic villus biopsy in a subsequent pregnancy and had a child unaffected by adenylosuccinase deficiency. This report adds vacuolating leukodystrophy as a novel feature of adenylosuccinase deficiency and shows the power of biochemical investigations directed by genomic studies to achieve accurate diagnosis. Importantly, this case demonstrates the importance of anticipatory banking of biological samples for reverse biochemical phenotyping in individuals with undiagnosed disorders who may not survive.

## 1 INTRODUCTION

Next‐generation sequencing has transformed the diagnosis of genetic conditions. However, interpretation of variants of uncertain significance (VUS) remains a major challenge. Reverse phenotyping through clinical history, examination, imaging or functional studies can help classify VUSs (De Goede et al., 2016; Landini et al., 2020). However, reverse phenotyping can be challenging in prenatal settings, or of very young or deceased individuals. Here, we present a case of an infant with adenylosuccinase deficiency (OMIM 103050) that expands the clinical spectrum of this rare disease and shows the value pre‐mortem banking of a range of tissue samples for anticipatory reverse phenotyping from individuals whose demise is expected.

## 2 CASE REPORT

### 2.1 Clinical presentation

**FIGURE 1:**
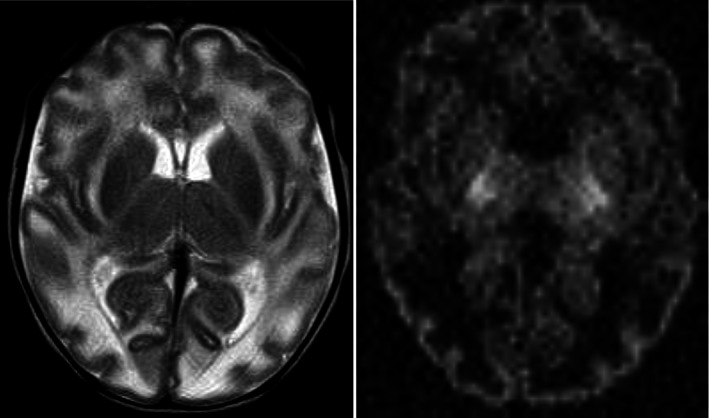
Clinical features and results of investigations. Magnetic resonance imaging of the brain findings at 14 days of age. The left panel axial T2 WI image shows high T2 signal in the cerebral white matter reflecting vasogenic oedema. No myelin deposition is seen in the posterior limbs of internal capsules bilaterally. The right axial DWI (diffusion‐weighted imaging) image demonstrates diffusion restriction in the thalamo‐capsular regions bilaterally in keeping with acute cytotoxic oedema.

The proband (male) was the first child of a nonconsanguineous couple of white British ethnicity with a previous early miscarriage of unknown cause. Excess in utero fetal movements were noted during pregnancy. The child was born by emergency Caesarean section, after induction at 41 weeks and 4 days of gestation with his APGAR scores of 6 at 1 min and 9 at 5 min. His birth weight was 2390 g (0.45 SD) and head circumference was 33 cm (1.24 SD). The newborn was noted to be severely hypotonic, with absence of spontaneous movements, and had a protruding tongue and bilateral deep creases between the great and the first toes. On Day 2 of life, he developed seizures, which were predominantly myoclonic jerks. He had several seizures per day and several episodes of hypothermia and hypoglycaemia. Seizures were refractory to treatment with levetiracetam, phenytoin, pyridoxal phosphate, vigabatrin, phenobarbitone, and benzodiazepines. The child's clinical features were suggestive of severe epileptic encephalopathy. Electroencephalogram showed burst suppression. Magnetic resonance imaging (MRI) of the brain obtained at 14 days of age showed signal abnormality of the entire cerebral white matter and to a lesser extent in the cerebellar hemispheres (Figure 1). The signal abnormality extended to involve external capsules and the claustrum bilaterally. Diffusion restriction was noted in the posterior limbs of the internal capsules bilaterally extending into the dorsal brainstem. Reduced diffusion was also seen in the adjacent lateral thalami bilaterally. There was no evidence of myelin deposition in the posterior limbs of the internal capsules bilaterally. Susceptibility weighted imaging sequences showed focal micro‐hemorrhages in the right occipital sub‐cortical white matter. The corpus callosum was diffusely thin in caliber and the temporal horns of the lateral ventricles appeared prominent. These results suggested a likely leukodystrophy, but its pattern and biochemical investigations (Table S1) did not lead to detection of the underlying cause. He developed pan‐enteric necrotizing enterocolitis on Day 51. Due to continued deterioration and extremely poor prognosis, he was too unstable for surgical management and after discussion with family, he was extubated electively and died within a few hours, at 52 days of age. As accurate diagnosis was not known, skin fibroblasts, skeletal muscle, plasma, and cerebrospinal fluid samples were stored. Parental consent was taken to gather samples.

### 2.2 Post‐mortem investigations

Macroscopically the brain had a marked lack of differentiation between the white and gray matter. White matter within the brain stem, cerebellum, and cerebrum showed an unusual vacuolar appearance. These findings in combination with the clinical features suggested a vacuolating leukoencephalopathy. A clinical exome analysis was performed on stored DNA sample as described previously (Patricia Molina‐Ramírez et al., 2021; Stoyle et al., 2018). This revealed two *ADSL* c.632T>A (p.(Leu211His)) and c.1277G>A (p.(Arg426His)) (NM_000026.2) variants that were confirmed to be in trans by bi‐directional Sanger sequencing of parental samples. Bi‐allelic loss‐of‐function *ADSL* variants cause adenylosuccinase deficiency (Georges & Berghe, 1984; Jurecka et al., 2015; Stenson et al., 2020; Stone et al., 1992). Several patients with the p.(Arg426His) variant have been previously reported (Donti et al., 2016; Mao et al., 2017) and this variant was, therefore, classed as pathogenic according to the American College of Medical Genetics and Genomics (ACMG) criteria (PS1 PS3 PM1 PM2 PP3; Richards et al., 2015). At the time of the discovery, the p.(Leu211His) variant was novel and classed as a VUS (PM1 PM2 PP3). As the variant was proven to be in trans with a pathogenic variant, it could potentially be reclassified to likely pathogenic. However, vacuolating encephalopathy has never been described with this condition previously.

### 2.3 Reverse biochemical phenotyping

Adenylosuccinase (EC 4.3.2.2) catalyzes two steps involving β‐elimination of fumarate in the de novo synthesis of purine nucleotides, converting succinylaminoimidazole carboxamide ribotide into aminoimidazole carboxamide ribotide (Kmoch et al., 2000). ADSL is also involved in the purine nucleotide cycle by forming adenosine monophosphate (AMP) from succinyladenosine monophosphate, which prevents AMP accumulation after adenosine triphosphate catabolism (Swain et al., 1984). Adenylosuccinase deficiency results in presence of succinylaminoimidazole carboxamide riboside and succinyladenosine (S‐Ado) in cerebrospinal fluid and plasma (Georges & Berghe, 1984). Following the genetic results, purine and pyrimidine metabolites were separated in previously stored plasma samples from the proband and quantitated by reversed‐phase UPLC with diode array UV detection on a Waters Acquity UPLC system (Waters). This showed elevated S‐Ado (1398.7 μmol/L) and SAICAr (696.0 μmol/L). Next, adenylosuccinase activity was measured in stored fibroblasts as described previously (Bierau et al., 2011) and was found to be 0.013 nmol/(μg prot × h) which was only ~5% of control. In ACMG classification, PP4 can be used as a supporting piece of evidence when the patient's phenotype is in its entirety consistent with a specific genetic etiology. These results could, therefore, be used to reclassify the VUS as likely pathogenic and thus confirming the diagnosis of adenylosuccinase deficiency in the child.

### 2.4 Subsequent pregnancy

Following the confirmation of the diagnosis in their deceased child, the parents opted for genetic testing via chorionic villus biopsy in the next pregnancy, which showed the fetus to be unlikely to be affected by adenylosuccinase deficiency. They now have a healthy 2‐year‐old child.

## 3 DISCUSSION

Collectively, the clinical and post‐mortem features along with the radiological, genetic, and biochemical findings confirmed a diagnosis of adenylosuccinase deficiency in the proband. The fatal neonatal form of the condition is characterized by variable combinations of impaired intrauterine growth, decreased fetal movements, loss of fetal heart rate variability, neonatal‐onset encephalopathy, microcephaly, intractable seizures, absence of spontaneous movements, respiratory failure, and death within the first weeks of life (Jurecka et al., 2015). In addition to the fatal neonatal form, ADSL deficiency is known to occur in Type I and Type II forms (Jurecka et al., 2015). Type I, the most common form, is characterized by onset within first few months of life with severe psychomotor delay, seizures, developmental arrest, severe cortical visual impairment, and microcephaly. Type II is a moderate or milder form of the disease and is characterized by onset within the first years of life with mild‐to‐moderate psychomotor delay, seizures, and ataxia in some patients. We expand the known phenotype spectrum of the condition by demonstrating vacuolating leukodystrophy in an individual with neonatal‐onset adenylosuccinase deficiency. Notably, spongiosis has been previously described in adenylosuccinase deficiency (Mierzewska et al., 2009). The early onset of vacuolization in a fatal neonatal case is a novel finding for this condition and reflects the severity of the defect as supported by the biochemical results. Of note, the white matter vacuolation was not seen in the MRI performed at Day 14 potentially suggesting of the progressive nature of the disorder. Additionally, the mother reported excessive fetal movements in contrast to usually found reduced movements. This could, therefore, be another feature of ADSL deficiency where the onset of seizures occurs prenatally, as demonstrated in this case.

This report shows the power of combined biochemical and genomic studies in molecular autopsies and in accurate diagnosis of inborn errors of metabolism (Ghosh et al., 2017). This was enabled by the previously banked plasma samples and fibroblasts from the deceased child. Without these samples, it might not have been possible to confidently give the diagnosis because vacuolating leukodystrophy is not a known feature of adenylosuccinase deficiency. Prenatal diagnosis, in the next pregnancy, therefore, may not have been possible. Reverse phenotyping has an important role in correlating variants with clinical features (De Goede et al., 2016). However, in deceased individuals, reverse phenotyping can be challenging and can limit the ability of diagnostic laboratories in providing prenatal or cascade testing. This case demonstrates the importance of having appropriate consent and anticipatory banking of biological samples for future reverse phenotyping in individuals with undiagnosed disorders who may not survive. Clinicians should consider this possibility, especially with increasingly earlier application of WES in the diagnostic pathways of neonates with unexplained severe diseases.

## AUTHOR CONTIBUTIONS

Grace Vasallo, Julija Pavaine, Lydia Bowden, Bernd Schwahn, and Siddharth Banka provided clinical details. Adele Fairclough and Ronnie Wright performed genetic analysis. Hetalika C. Banka, Lynnette Fairbanks, Jörgen Bierau, and Alistair Horman performed biochemical analyses. Spatikha Sitaram, Hetalika C. Banka, and Siddharth Banka wrote the article. All co‐authors read and approved the manuscript.

## Supporting information


**Table S1** Extensive biochemical investigations and results

## Data Availability

The data that supports the findings of this study are available in the supplementary material of this article.
