# Supplementary material for: Anticipatory banking of samples enables diagnosis of adenylosuccinase deficiency following molecular autopsy in an infant with vacuolating leukoencephalopathy
Source: Am J Med Genet A. 2022 Oct 22;191(1):234–7. doi: 10.1002/ajmg.a.62999 (PMC10091700; doi:10.1002/ajmg.a.62999)
Supplement: Supplementary file 1 — Table S1 Extensive biochemical investigations and results [file AJMG-191-234-s001.docx]

**Supplementary information**

**for**

**Anticipatory banking of samples enables diagnosis of adenylosuccinase deficiency following molecular autopsy in an infant with vacuolating leukoencephalopathy**

**Supplementary Table S1**

| **Extensive Biochemical Investigations** | **Results** |
| --- | --- |
| Urine Analysis | No evidence of organic aciduria |
| Urine sulphite and sulphocysteine | Negative |
| CSF | Lactate, glycine, amino acids, glucose, protein, plasma amino acids all normal |
| Very long fatty acid analysis | Slight increase in C24/22 and C26/22 ratio |
| Blood spot acylcarnitines | Normal, no significant increase in free carnitine |
| Biotininidase activity | Normal |
| Mitochondrial enzymes – complexes (I-IV) | Normal |
| Fibroblast pyruvate dehydrogenase (PDH) | Normal |
| TSH | 1.77 (Reference Range: 0.27-4.20mU/L) |
| Free T4 | 6.3 (Reference Range: 15-34 pmol/L) |
